# Nanoemulsions: A Review on the Conceptualization of Treatment for Psoriasis Using a ‘Green’ Surfactant with Low-Energy Emulsification Method

**DOI:** 10.3390/pharmaceutics13071024

**Published:** 2021-07-06

**Authors:** Ignatius Julian Dinshaw, Noraini Ahmad, Norazlinaliza Salim, Bey Fen Leo

**Affiliations:** 1 Department of Chemistry, Faculty of Science, University of Malaya, Kuala Lumpur 50603, Malaysia; ignatius@um.edu.my; 2 Integrated Chemical Biophysics Research, Faculty of Science, Universiti Putra Malaysia, Serdang 43400, Malaysia; 3 Nanotechnology & Catalysis Research Centre (NANOCAT), Institute of Advanced Studies, University of Malaya, Kuala Lumpur 50603, Malaysia; beyfenleo@um.edu.my; 4 Faculty of Medicine, University of Malaya, Kuala Lumpur 50603, Malaysia

**Keywords:** psoriasis, anti-psoriatic agent, nanoemulsion, biosurfactant, low-energy emulsification

## Abstract

Psoriasis is a skin disease that is not lethal and does not spread through bodily contact. However, this seemingly harmless condition can lead to a loss of confidence and social stigmatization due to a persons’ flawed appearance. The conventional methods of psoriasis treatment include taking in systemic drugs to inhibit immunoresponses within the body or applying topical drugs onto the surface of the skin to inhibit cell proliferation. Topical methods are favored as they pose lesser side effects compared to the systemic methods. However, the side effects from systemic drugs and low bioavailability of topical drugs are the limitations to the treatment. The use of nanotechnology in this field has enhanced drug loading capacity and reduced dosage size. In this review, biosurfactants were introduced as a ‘greener’ alternative to their synthetic counterparts. Glycolipid biosurfactants are specifically suited for anti-psoriatic application due to their characteristic skin-enhancing qualities. The selection of a suitable oil phase can also contribute to the anti-psoriatic effect as some oils have skin-healing properties. The review covers the pathogenic pathway of psoriasis, conventional treatments, and prospective ingredients to be used as components in the nanoemulsion formulation. Furthermore, an insight into the state-of-the-art methods used in formulating nanoemulsions and their progression to low-energy methods are also elaborated in detail.

## 1. Introduction

The term psoriasis is derived from the Ancient Greek word ‘psora’, which means itch. In ancient times, psoriasis was generalized with contagious inflammatory skin disorders such as leprosy [1]. However, psoriasis is a chronic non-contagious hereditary skin disease that causes severe itching. The condition causes the skin to be inflamed, thickened, scaly, and deformed [2]. Its recorded occurrences around the globe give statistics of 2–5% [3,4,5]. Psoriasis manifests in various forms, and its classification is made on the basis of the inflammatory process undergone, localization of rash, local irritation, the severity of occurrence, and other clinical traits. This disease has four categories: chronic plaque, guttate, pustular, and erythroderma [6]. Most cases reported are of chronic plaque psoriasis in which both sexes are equally affected in their early 40s [7]. Since psoriasis is a prolonged condition, it calls for a long-term treatment. Among the anti-psoriatic drugs used are retinoids, methotrexate, and cyclosporine. However, these drugs incur numerous risks of side effects such as lip inflammation, hair loss, stomach-ache, liver damage, and kidney problem [2,8].

Topical psoriatic treatments such as emollients, coal tar, and dithranol are safer but have shown low efficiency and are cosmetically indifferent [9,10]. Modern medicine has explored many approaches to treat this illness (e.g., oral intake of drugs, injections, and steroid-based creams), but all these methods fall short as they do not have a safe and optimal carrier that can deliver the anti-psoriatic drug effectively for maximum therapeutic effect [5,11]. To enhance the efficiency of anti-psoriatic drug delivery and reduce its adverse side effects, researchers have implored nanotechnology to address the limitations of conventional treatments. Nanosized drug delivery systems have immense potential to enhance drug delivery and reduce prescribed dosages due to their increase in bioavailability. Nanoemulsion is one such technique that uses a colloidal model to deliver nanosized droplets of active agents with high surface area to the affected region of skin.

Nanoemulsion can be defined as a kinetically stable clear dispersion of two immiscible phases (oil phase and water phase) in combination with surfactant molecules with droplet sizes ranging from 5 to 200 nm [12,13]. The use of nanoemulsion as a carrier for anti-psoriatic drugs is advantageous as there is no intrinsic creaming, flocculation, sedimentation, or coalescence, which are commonly observed in macroemulsions [14]. In terms of topical delivery, nanoemulsions do not cause skin irritation, have high permeation ability, and have high drug-loading capacity [5,15]. In the preparation of an optimal nanoemulsion, the selection of suitable oils and surfactants are key. In this review, the prospective use of biosurfactants in place of their synthetic counterparts is highlighted in terms of cost-effectiveness, sustainability, and environmental compatibility. The term biosurfactant indicates that the surface-active agent is from a biological source and therefore offers a ‘greener’ alternative to its chemically derived counterparts. Biosurfactants are also less toxic and function well in extreme pH and temperatures. Most importantly, these biosurfactants are produced from bacteria, making them sustainable, as bacterial growth is fast and supply is replenishable and abundant [16].

In this review, the psoriatic-pathogenic pathway, conventional treatments, and the move towards a nano-sized topical approach is discussed. Moreover, an insight on the current nanoemulsion methods used in formulation and its progression to low-energy methods are elaborated in detail. An overview on the suitable ingredients to be used for a nanoemulsion and the introduction of the use of natural biosurfactants as opposed to the synthetic ones are highlighted as they are environmentally friendly and exhibit surfactant competency on par with their synthetic counterparts.

## 2. Pathogenic Pathways of Psoriasis

**Figure 1:**
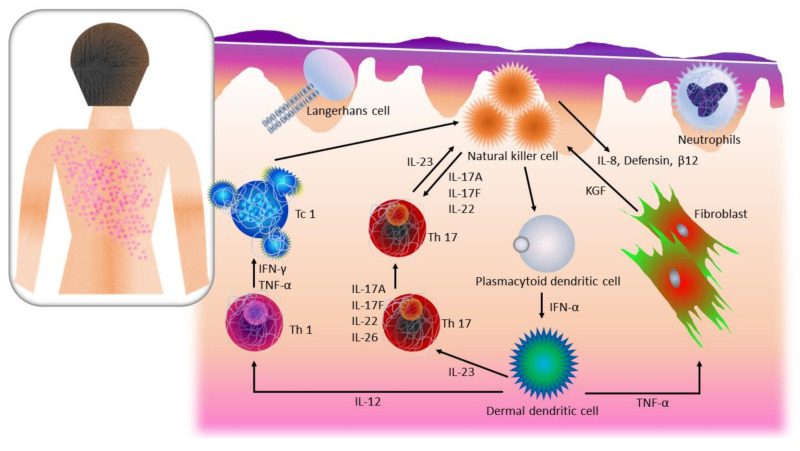
The flowchart of the pathogenic pathway of psoriasis. This figure is self-drawn.

The first step in proposing a possible solution for psoriasis is to have a better understanding of the molecular processes involved in the psoriatic condition. Psoriasis is known to occur via the most accepted hypothesis through several pathogenic pathways. In general, each of these pathways has components that when triggered transmit information to either initiate a response or block it. These components include T cells, antigen-presenting cells (APCs), keratinocytes, Langerhans’ cells, macrophages, natural killer cells, an array of Th1-type cytokines, transcription factor NFκB, and others. Briefly, the pathogenic reaction is initiated by the activation of the T cells by the release of antigens. This leads to the secretion of cytokines, inflammatory cells, and keratinocytes. The characteristic hardening and scaling on post-psoriatic skin are due to the over-proliferation of the keratinocyte [17]. An overview of the pathogenic pathway of psoriasis is illustrated in Figure 1.

### 2.1. T Cell Activation, Cytokine Release, and Over-Proliferation of Keratinocytes in Psoriasis

T cells, are also known as T lymphocytes, are the main component of an adaptive immunoresponse. The function of these cells is to initiate an immunoresponse to fight against pathogens that have invaded the body and provide information through antibody response for protection from pathogens outside the body [18]. T cells also act against self-antigens that are initiated by dendritic cells in the dermis at the early stages of psoriasis [19]. As the psoriatic episode worsens, dermal dendritic cells mature and initiate production of tumor necrosis factor α (TNFα) and interleukin-23 (IL-23) that activate autoreactive CD8+ T killer cells, and some CD4+ T helper cells move towards T helper 17 cell (Th17) phenotype or the interleukin IL-17+interferon γ (IFNγ)+ pathogenic Th1/Th17 [19,20,21,22]. T cell activation can also move to the skin epidermis and recognize epidermal autoantigens to initiate the differentiation of tissue-resident memory T cells (TRM) [23,24]. Epidermal autoantigen recognition by cytotoxic T cell 1 and 17(Tc1/Tc17) initiates the release of cytokines including IL-22, causing early epidermal hyperproliferation and activation of keratinocytes. A hyperproliferating keratinocyte cell cycle in normal conditions takes four days, whereas in psoriatic cases, it may stretch up to a month [25]. This prolonged hyperproliferation will initiate the release of antimicrobial peptides and chemokines [26,27,28,29].

### 2.2. Natural Killer Cells in Psoriasis

Natural killer (NK) and NK-T cells are vital producers of inflammatory cytokines, indicating that these cells are the main factors in a psoriatic episode. NK cells are the main component of the innate immune response to viral infection and tumor growth [30]. These cells are activated by IFN released by dendritic cells (DCs) and tumor necrosis factor (TNF) upon stimulation of toll-like receptors (TLRs), commonly TLR3, TLR7, or TLR9, which are activated by nucleic acids present in viruses [31]. Upon activation, NK cells serve as a containment to capture the viral infections and normally dispose of them without alerting the adaptive immunoresponse. They function also as producers of large amounts of cytokines, in particular IFN and IL-4, and when activated by DCs, these cells act synergistically to influence the adaptive immunoresponse. NK cells also produce β-defensins and cathelicidins, which are antimicrobial peptides that cause overexpression in a psoriatic episode [31,32]. Once NK cells reach the skin, they produce IFN, which has been shown to cause Th17 cell accumulation on the affected skin region [32]. NK-T cells are lymphocytes that express NK receptors, but unlike NK cells, they also express T cell markers including cluster of differentiation 3 (CD3) and T cell receptors (TCR) α and β [33]. NK-T cells are like NK cells and may incur cytotoxicity and/or immunoregulatory response. NK-T cells can produce a significantly higher amount of cytokine production than NK cells, including secretion of IL-4, IL-5, IL-10, IL-17, and IFN. A NK-T subgroup known as invariant NK-T cells also expresses the restricted T cell receptor Vα24Vβ11. These cells are activated as glycolipid antigen from CD1d molecule is recognized. This antigen-presenting molecule has been found to be overexpressed by keratinocytes during the inset of psoriasis [34]. The immunoresponse in psoriasis is similar to a viral infection, and therefore this pathway can help to explain the psoriatic inset from a different viewpoint [35].

### 2.3. Nuclear Factor-Kappa B in Psoriasis

Nuclear factor-*kappa* B (NF-ĸB) is a protein transcription factor that initiates inflammation and other complex biological processes. It is a main regulatory component in immune and inflammatory pathways, cell differentiation, proliferation, and apoptosis; hence, it is a vital mediator in the psoriatic pathogenic pathway. NF-ĸB modulates keratinocyte and immune cell states through its effects on cell proliferation, differentiation, and apoptosis, including chemokine and cytokine production [36]. Components of the innate immune system, such as TLR2 [37] and caspase-5 [38], recognize pathogen-associated molecular patterns (PAMPs) and are upregulated at the onset of psoriasis, depending on the NF-ĸB returning to normal levels through indirect activation of pro-inflammatory cytokines. As the process continues, apoptotic signals decrease in the epidermal keratinocytes, altering cell death rate, which causes the epidermal cell cycle to prolong nine times longer than usual [39]. The altered NF-ĸB signaling regulates apoptotic signals such as B-cell lymphoma-extra-large gene (Bcl-xL), cyclins, and survivin, which are known to be at peak in an inset of psoriasis [40,41].

### 2.4. Langerhans’ Cells in Psoriasis

Langerhans’ cells are a subset of dendritic cells residing in the epidermis. The function of Langerhans’ cells is to constantly monitor the external environment and report to the T cells by delivering signals and/or antigens. In a psoriatic episode, Langerhans’ cells would capture the invading pathogen and become activated and migrate to the draining lymph node and present the antigen to T cells to elicit immunoresponse [42]. Langerhans’ cells in human skin may also activate Th2 and Th22 cells [43].

## 3. Current Psoriatic Treatments

Psoriatic treatments are key in keeping the illness at bay or at least bearable enough for the affected individual. Psoriasis treatments help in stopping the itching and reduce inflammation. The existing treatments for psoriasis, both synthetic and their respective mechanism, are as shown in Table 1. Conventional drugs can be administered into the body both topically and systemically, depending on the severity of the case. In a major case, the systemic route is commonly chosen to relieve the itching almost instantly, as the drugs prescribed generally hinder the pathogenic pathway and in turn stop the inflammatory response. The mode of action of systemic drugs mostly function to suppress the autoimmune system by inhibiting the signaling molecules from transmitting information through the pathway. For the topical approach, prescribed drugs would be administered on the skin surface to inhibit autoimmune system component on the epidermis by acting as the immunomarker to inhibit the over-proliferation of skin cells and encourage cell differentiation. Comparatively, topical drugs are more user-friendly than systemic drugs, as they are administered superficially and not ingested. However, topical drugs are commonly prescribed for mild to moderately severe cases, and as soon as this threshold is reached, systemic drugs are the better option due to inadequate drug penetration into the skin. Natural remedies are a traditional approach and may be beneficial to the mild inset of psoriasis as it is believed to have no side effects. The disadvantage of consuming systemic drugs is its decrease in efficiency over time because of the production of anti-drug antibodies. Furthermore, long-term use of these drugs has evidently led to organ poisoning, tumor formation, and non-melanoma skin cancer [44]. For this reason, a topical nanoemulsion would serve as a good alternative as it is a superficial treatment with enhanced bioavailability due to minuscule particle size. The mode of action of topical drugs is to inhibit the cell proliferation that contributes to the skin thickening and darkening (post-psoriasis effect). The topical drugs also initiate cell differentiation to help the skin regain its original form. Some topical drugs possess antibacterial properties to ward off skin microflora from infecting the damaged skin

Table 1 summarises the general topical drugs used (i.e., betamethasone, calcipotriol and tacalcitol) that regulate the immune response from the skin surface. The use of betamethasone and calcipotriol in combination was seen to have high potential for long term use as seen in an extensive study done by Luger et al. where a double-blind study was performed on 869 patients with scalp psoriasis. In this study, the adverse effect of corticosteroid usage was less seen in combination than just calcipotriol [45]. There are studies where betamethasone is administered individually, and it has shown positive outcome in undermining dermatitis in the elderly [46]. Tacalcitol has shown positive results when paired with narrow-band ultraviolet B treatment [47]. Amongst these drugs, betamethasone seems like the best candidate to be incorporated in a treatment individually whilst taking advantage of nanotechnology to enhance its therapeutic effect.

## 4. Nanoemulsions as a Topical Drug Delivery System

Upon understanding the mechanism of action of conventional drugs, a discussion can be struck as to which mode of consumption the patient is to be treated with the intended treatment. In general, a topical approach would be a less invasive method that would receive high compliance from patients, and since this review addresses psoriasis, a skin ailment, it is most apparent. In terms of which nanodrug delivery system is best to be used for this purpose, a brief comparison has to be made between all existing nanosystems to come to a decision. Nanosystems are a step forward from microsystems as the nano-approach increases the efficiency of drug activity. By reducing the particle size, bioavailability increases in turn, reducing the amount of drug needed to be administered into or onto the patient. This reduces the potential amount of toxin build-up in the body, which in turn helps undermine possible adverse side effects. Reduction in dosage also reduces the demand for excessive production through the supply chain whilst still catering to the target group. The nano-sizing of the colloidal system also helps in the miscibility of low solubility drugs [88]. Localization of drugs for area-specific conditions are made possible through this approach [89]. In some cases, nanoparticles are able to transport large amounts of drug to an affected group of cells whilst sparing the unaffected cells [90]. The limitation of this approach lies in the unknown nanotoxicity, which precedes the treatment that cannot be detected using conventional facilities. This would be an area upgrade as the technology develops further [91].

Nanosystems are present in many variants, namely, nanoemulsions, biopolymeric nanoparticles, liposomes, polymeric micelles, dendrimer, and quantum dots [92]. Nanoemulsions are a colloidal system that is in the range of 10 to 1000 nm. They have a structure of a solid sphere with an amorphous surface, which is lipophilic [93]. Liposomes are vesicles that range from 30 nm to several micrometers, consisting of monolamellar or multilamellar bilayers of steroids and phospholipids. These techniques use encapsulation to load in lipophilic and hydrophilic drugs. Micelles are sphere structures of the size range of 10 to 100 nm with amphiliphilic-block polymers made up of polyethylene glycol hydrophilic corona and a lipophilic core. Micelles’ mode of uptake is by solubilizing lipophilic drugs into the core. Niosomes are vesicles like liposomes that are made up of non-ionic surfactants with lamellar or unilamellar bilayer structures. Niosomes are able to uptake lipophilic and hydrophilic drugs by the hydration dried non-ionic surfactant in which the drugs are trapped both in the aqueous layer and vesicles. Biopolymeric nanoparticles have a size range of less than 100 nm and comprise an array of different forms, among them, nanoshells, quantum dots, and fullerenes. These nanoparticles have different modes of uptake depending on their release characteristics and chemical properties. In general, nanoparticles form a matrix-like structure known as a nanosphere. Some examples of commonly used nanoparticles include chitosan, alginate, xanthan gum, and cellulose. Dendrimers are macromolecules with branched structures stemming from a central core. Dendrimers’ unique feature is their branched structure, which allows for drug transportation both through their internal structure and externally attached [94]. In general, a nanoemulsion system is advantageous as it has a lower production cost and lesser process complexity in comparison to other nanosystems [89]. It is also been seen that nanoemulsions more efficiently transport lipophilic drugs than liposomes due to their lipophilic interior [14]. Nanoemulsions are also feasible to be scaled up to larger productions and also manipulated into various forms, for example, creams, liquids, and aerosols [95].

### 4.1. Definition of Nanoemulsion

The IUPAC definition of emulsion is a fluid colloidal system in which liquid droplets and/or liquid crystals are dispersed [96]. The combination of nanotechnology in emulsion gives it a new form, to which utmost attention is given to the nanosize of the liquid droplet. Nanoemulsions can also be defined as a thermodynamically stable transparent nanosized dispersion of oil in water (O/W) or water in oil (W/O), with an interfacial component of surfactant and cosurfactant molecule that stabilizes the system with the droplet size 10–100 nm [97]. Several studies have reported discrepancies in the acceptable range of nanosize in nanoemulsions. Shah and Bhalodia defined the nanoemulsion range at 5–200 nm [98]. Shakeel and co-workers, on the other hand, defined nanoemulsion range at 10 to 140 nm on the basis of their experimental assessment [99]. In another study, Mason and co-workers set the range at 20 and 200 nm [15]. The studies were unified to obtain the desired range of 5–200 nm.

### 4.2. Components of Nanoemulsion

**Figure 2:**
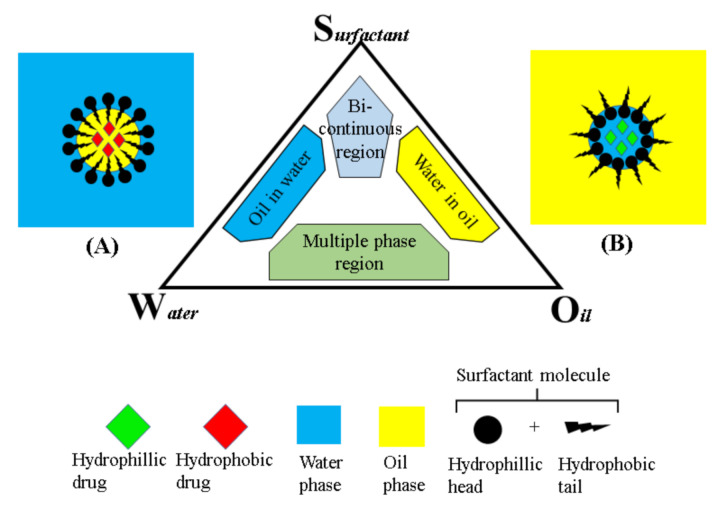
The phase diagram illustrates the components of nanoemulsions; oil, surfactant, and water. The schematic diagrams (**A**,**B**) represent oil-in-water and water-in-oil nanoemulsions, with the surfactant molecule positioned oppositely in each case. This figure is self-drawn.

Nanoemulsion is generally made up of two phases: The oil phase and the aqueous phase (Figure 2). The oil phase may be developed from a mixture of oils or a single oil that is infused with the active ingredient of choice. Commonly, the oil phase base is made up of triacylglycerols, monoacylglycerols, diacylglycerols, and free fatty acids and it is incorporated with desired drugs and lipophilic surfactants. The aqueous phase is mostly composed of water with surfactants, cosurfactant, emollient, and other components [100]. The nanoemulsion produced varies depending on the oils, surfactants, and other components used in the mix; however, the oil chosen majorly affects the formation, functional properties, and stability of the nanoemulsion [101,102,103]. Upon combination of the oil phase and aqueous phase, a phenomenon known as Ostwald ripening may occur, which is generally the increment in droplet size as opposed to time. Other forms of degradation may occur, as well such as coalescence, gravitational separation, or flocculation [104]. In order for this occurrence to be prevented, stabilizers are introduced into the system. These stabilizers are mainly preservatives, thickening agents, and emulsifiers, which serve to ensure the integrity of the nanoemulsion. As oil and water are at an interface, they can assume four different forms, oil-in-water (O/W), water-in-oil (W/O), bi-continuous or lamellar nanoemulsion, and multiple nanoemulsion [105,106]. An o/w nanoemulsion forms when an oil phase is dispersed in a continuous aqueous phase favored by a hydrophilic surfactant. A W/O nanoemulsion occurs when water droplets are dispersed in a continuous oil phase that is favored by a lipophilic surfactant [106]. A bi-continuous nanoemulsion usually forms when the volumes of oil and water are approximately the same, the phases then being inter-dispersible through the system. In the multiple emulsion system, water-in-oil-in-water (W/O/W) and oil-in-water-in-oil (O/W/O) emulsions may form. The W/O/W emulsions are made of large oil droplets containing water droplets dispersed in an aqueous phase. On the other hand, in the O/W/O emulsion system, water droplets containing oil droplets are dispersed in an oil phase [14,107].

### 4.3. Physicochemical Characteristics of Nanoemulsion

The selection of an appropriate oil for the oil phase is a key part of nanoemulsion formulation. Saturated and unsaturated fatty acids/fatty acid esters are commonly used as an oil phase as they enhance the therapeutic effect of certain drugs [108]. The chemical and physical characteristics play a vital role in the selection process. There are many parameters in which one may need to consider when producing a nanoemulsion specific to a certain application. Among these factors are particle size, polydispersity index, zeta potential, pH, viscosity, density, in vitro drug release, in vitro permeation, stability, thermodynamic stability, shelf life, refractive index, percent transmittance, pH, dispersibility, viscosity, surface tension, friccohesity, and osmolarity [109,110]. Some common factors such as particle size, polydispersity index (PDI), zeta-potential, hydrophilic–lipophilic balance (HLB), pH, density, and viscosity are discussed in this review.

The polydispersity index is a measure heterogeneity of size distribution of particles. The PDI values range from 0.0 to 1.0, where 0.0 indicates that the distribution is a perfectly uniform sample and 1.0 indicates that the sample is highly polydisperse with multiple particle size populations. PDI values of 0.2 and below are set as an acceptable value for nanoparticle in the field of polymers [110]. The zeta potential is a characterization technique for nanocrystals to estimate the surface charge, which can help better understand the physical properties of a nanodispersion. A high positive or negative zeta value indicates that there is an electrostatic repulsion keeping each particle apart. A low zeta value may indicate that the particles form an aggregate or flocculate due to Van der Waals forces drawing the particles together. Aside from zeta values ranging from −30 mV to +30 mV, all other values indicate that the dispersion has enough repulsion between particles to form a stable colloidal system [111].

The ideal pH for skin is between pH 4.0 and 7.0. A study revealed the effect of pH on skin microflora, wherein an acidic pH (4–4.5) did not harm the resident microflora on the skin, whereas an alkaline pH (8–9) caused the microflora to dispel from the skin [63]. Viscosity is another important factor—its function is to minimize the fluidity and give the nanoemulsion its final desired consistency. Viscosity plays a vital role in the stability and drug release of the nanoemulsion formulations. Surfactant, water, and oil components and their concentrations are what make up the viscosity of the emulsion. Higher water content in the emulsion will lead to a reduction in viscosity while decreasing the surfactant, and co-surfactant content will increase viscosity due to interfacial tension between the water and oil [112].

The hydrophilic–lipophilic balance (HLB) value is another driving factor to produce a stable nanoemulsion. HLB is the balance of the size and strength of the hydrophilic and lipophilic moieties of a surfactant molecule. In a low HLB surfactant condition, the system will assume a W/O emulsion form, while high HLB surfactants will take an o/w emulsion form. The HLB scale ranges from 0 to 20. The surfactant with HLB values ranging from 3.5 to 6.0 is normally used in a W/O emulsion. Surfactants with HLB values of 8 to 18 range are normally used in an O/W emulsion [113]. Another important factor in surfactant selection is their electrical charge, whether it is nonionic, zwitterionic, anionic, or cationic. This may affect the stabilizing mechanism of the polar head of the surfactant with the aqueous phase. The ionic surfactants are additionally stabilized by the electrostatic interactions, whereas nonionic surfactants are stabilized by dipole and hydrogen bond interactions with the hydration layer of water and by repulsive forces due to steric hindrance [114].

### 4.4. Selection of Oil Phase

There are numerous reports of various oils that have been used in the formulation of nanoemulsions (Table 2). A suitable oil should be chosen on the basis of the type of application the nanoemulsion is to be used for. For example, in cosmetics, plant-based oils are favored because they are generally non-hazardous and compatible with the skin. In a study by Esquerdo et al. [115], fish oil was used to develop a supplement for human consumption due to its rich unsaturated fatty acid (UFA) content. In order to increase its solubility in water, the researchers proposed a nanoemulsion model. The droplet size from this study was 96.5–166.5 nm. Katzer et al. [116] developed a castor oil and mineral oil nanoemulsion to be compatible with contact lenses. The droplet size achieved was 234 nm. Bhusal et al. [117] developed a nanoemulsion with corn oil, achieving a droplet size of 181–187.9 nm. Sungpud et al. [118] used coconut oil in their nanoemulsion infused with mangosteen extracts as a supplement. The nanoemulsion had a droplet size less than 100 nm. Other oils such as evening primrose [119], linseed [120], olive [121], palm [122], sunflower [123], neem [124], babchi [125], grapeseed [126], chaulmoogra [127], tea tree [128], argan [129], sesame [130], and jojoba [131] oils have also been used as an oil phase in nanoemulsions. There are several waxes that have been used as an oil phase in the formulation of nanoemulsion [132,133,134,135,136]. Some oils, such as babchi oil and neem oil, have potential synergetic qualities in the formulation of anti-psoriatic nanoemulsions as they have a history of curing skin illnesses. Traditional medicine commonly uses neem in the treatment of chicken pox, and this treatment has been retained through the years. Furthermore, azadirachtin is the active ingredient present in neem, and it has been listed in Table 1 as a natural treatment for psoriasis. Babchi oil is also suitable to be used against psoriasis as it has psoralen as an active ingredient. Psoralen is a photoactive furocoumarin that can form binding with DNA in UV exposure to form pyrimidine bases. Due to this, babchi oil inhibits the synthesis of DNA, thereby stopping the over-proliferation of skin cells at the inset of psoriasis [125]. All the oils and waxes that have successfully formed nanoemulsions in previous studies are summarized in Table 2.

### 4.5. Selection of Biosurfactants

Surfactants are materials that lower the surface tension of two immiscible liquids when they are in interphase with each other. Generally, surfactants are used for cleaning, wetting, dispersing, and emulsifying. Paints, detergents, sanitizers, and shampoos are some examples of surfactant application. There are four categories of surfactant, all classified on the basis of the polarity of the head molecule. If the head is neutral, the surfactant is termed as nonionic. If the head is negative, the surfactant is anionic, and if positive, it is known as cationic. If the surfactant comprises two opposing charges, it is termed zwitterionic. Ethoxylates, ethylene, sorbitan esters, and propylene oxide copolymer are examples of some of the nonionic surfactants available. Fatty acids, ester sulphonates, or sulphates are examples of anionic surfactants. Quaternary ammonium salts are examples of cationic surfactants [137]. Some commonly used nonionic surfactants include Tween 20, Tween 40, Tween 80, Cremophor RH 40, and polyethylene glycol monooleyl ether (Oleth-20).

Biosurfactants are a natural alternative to the commonly used synthetic surfactants. These molecules are neutral or anionic and are sourced from microbes and fungus. They have a moiety to accumulate between fluid phases thereby decreasing surface and interfacial tension [138]. Biosurfactants are less toxic and function well in extreme pH and temperatures. Most importantly, these biosurfactants are produced of bacterial progenation making them sustainable as growth time is fast and supply is replenishable and abundant [16]. In Table 3, a list of biosurfactants that are commonly used with their respective structures and properties. The biosurfactants are classified based on their chemical composition, thereby forming three groups, namely glycolipids, polymeric biosurfactants and lipopeptides and oligopeptides. Comparatively, glycolipids stand out from the rest as they have skin reparative qualities. As psoriasis is a skin condition where unbearable itching leads the affected person to scratch the skin aggressively, most of the work should be focus on clearing the post-psoriatic scarred skin. Glycolipids would be a suitable surfactant in an anti-psoriatic nanoemulsion as it has synergetic qualities of both stabilizing the nanoemulsion and healing the scarred skin. In comparison to synthetic surfactants, biosurfactants are less toxic to the environment, are biodegradable and have higher foaming capacity [139,140]. The importance of its biocompatibility can be seen in the application of biomediation, where hazardous waste in the earth is to be broken down without compromising the quality of soil [141]. In Table 4, a comparison of the colloidal properties of biological and synthetic surfactants are compiled. Based on the findings, biosurfactants had lower critical micellization concentration (CMC) values (1.04–130mg/mL) compared to the synthetic set (17–3000mg/L), therefore exhibiting their strength as a surfactant and reinforcing the idea that common synthetic surfactants might one day be substituted by these ‘greener’ alternatives.

## 5. Formation of Low-Energy Nanoemulsions

Nanoemulsions can be formed by using two methods (high energy and low energy). In high-energy techniques, three methods are commonly used: high-pressure homogenization, microfluidization, and ultrasonication. In these techniques, high energy is presented in the form of high pressure and sonication to break larger particles to nanosize. Temperatures also fluctuate as processing takes place due to the high shear force demanded by these methods. Nanoparticles produced by this method should be able to withstand abrupt heating and cooling temperatures without losing their structural and chemical integrity. A more suitable alternative to nanosized particles whilst maintaining their original physical characteristics would be to use low-energy methods. Low-energy methods include phase inversion emulsification method, self-nanoemulsification drug delivery system (SNEDDS), and D-phase emulsification (listed in Table 5).

### 5.1. Phase Inversion Emulsification

**Figure 3:**
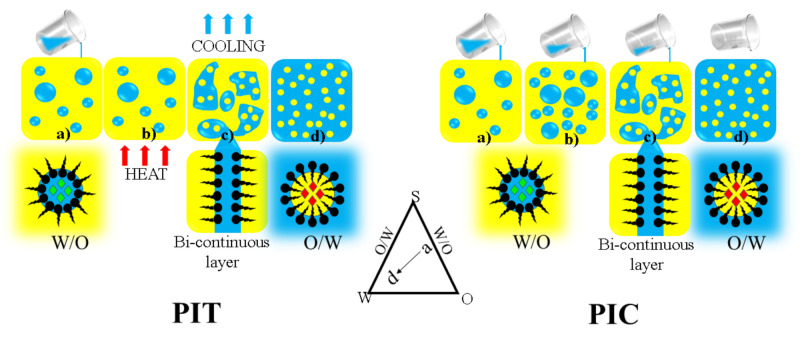
Illustration of phase inversion emulsification with varying temperatures (PIT) and varying compositions (PIC). The emulsion undergoes steps a-d to finally form a O/W emulsion. This is a self-drawn figure.

The phase inversion emulsification method stems from the phenomenon of agitating oil and water and the way their phases shift from oil to water (O/W) and water to oil (W/O). This method can be categorized into two methods: transitional phase inversion methods (TPI) and catastrophic phase inversion methods (CPI). TPI occurs when there is a change in the affinity of a surfactant or spontaneous curvature in the system leading to changes in temperature and composition. CPI is based on a gradual dilution of water or aqueous phase into the oil phase at a slow flow rate in which a nanoemulsion is produced. TPI can be further classified into two categories: phase inversion temperature (PIT) and phase inversion composition (PIC), wherein the names refer to the shift in phases in terms of the change in temperature and composition, respectively (Figure 3) [185]. Experimentally, in PIC method, an oil phase and an aqueous phase are homogenized in a beaker at room temperature. Different ratios of a mixture of oils, surfactants, cosurfactants, and other component are evaluated until an optimal concentration is established for long-term stability. Finally, water is added into the mixture at a slow gradual pace following the set concentrations, and thereafter, the product is subjected to minimal agitation at low speed until a stable emulsion is visible [186]. In the case of PIT, the compositions of the system are kept constant, and the varying factor is the temperature. In CPI, gradual addition of an aqueous phase into an oil phase is done with a slow flow rate until the emulsion inversion point (EIP) is reached, forming a nanoemulsion [187]. The term catastrophe in CPI refers to a sudden change in the behavior of the overall system when the water phase is added to the oil phase forming a W/O nanoemulsion. Once the critical water content is exceeded, the particle will start to merge, indicating that the phase inversion point has been reached. This phenomenon will cause a bi-continuous or lamellar structure to form. Further increase in water content will cause the phases to invert from a W/O to an O/W system [188]. In a study by Maestro et al., the authors utilized the PIC method to produce a hexadecane and oleic acid mixture nanoemulsion. In this study, an anionic surfactant was used, and it was observed that in order to reach a nanosize droplet, the mixture must remain in a direct cubic liquid crystal phase for enough time with adequate mixing during emulsification [189]. Jaworska et al. used a PIC method to produce caprylic/capric triglycerides and oleic acid nanoemulsion, wherein polysorbate 80 served as the surfactant. The nanoemulsion had a particle size of 25 nm [175]. Ragelle et al. reported the use of PIT in producing a nanoemulsion of a natural flavonoid fisetin in a miglyol and Tween 80 mixture system. They managed to achieve a droplet size of 153 nm [176]. In a study by Mashhadi et al., a lemon oil nanoemulsion was produced using PIT method with Tween 40 as the surfactant. The droplet size for this nanoemulsion was 9.6 and 11.1 nm. Chuesing et al. produced a cinnamon oil nanoemulsion using PIT method with Tween 80 as surfactant. The mean droplet size for this nanoemulsion was 101 nm [177].

### 5.2. Spontaneous Emulsification

**Figure 4:**
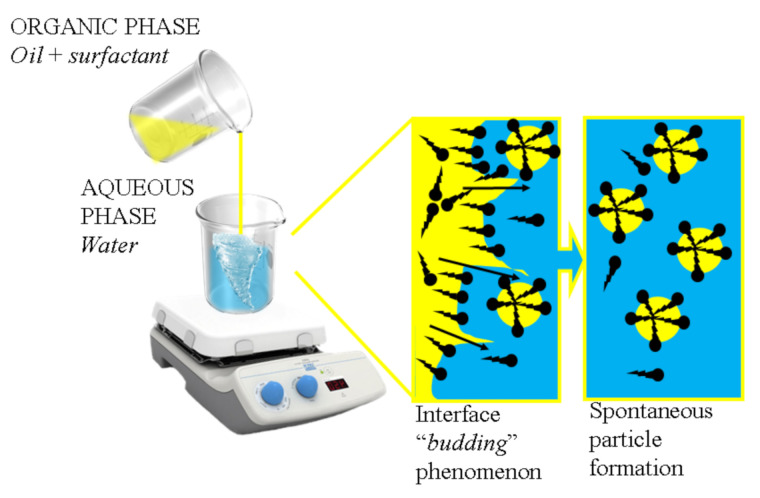
Illustration of the spontaneous emulsification mechanism. This is a self-drawn figure.

Spontaneous emulsification is another commonly used low-energy method. This technique begins by producing two phases that are present in non-equilibrium conditions: one consisting of an oil phase with a lipophilic surfactant, and another of water-miscible solvent and hydrophilic surfactant (Figure 4). The driving force in this method is the gradient of chemical potential between the phases. Under a certain condition, the chemical potential between the two phases may in some conditions produce negative values of free energy of emulsification [13]. The two phases are incorporated by injecting the oil phase into the aqueous phase coupled with continuous stirring. After the mixing process, the mixture is subjected to low-pressure evaporation to remove the aqueous phase. At this point, tiny droplets of the dispersed phase coated with surfactant forms in the continuous phase due to the surfactant’s high affinity to the oil phase. Interfacial turbulence at the interphase of the dispersed phase and continuous phase will determine whether the surfactant displaces into the continuous phase.

To ensure the emulsion is in nanosize, interfacial turbulence of the phases can be initiated by using cosurfactants such as acetone, ethanol, and propylene glycol [190]. This method produces nanosized particles as a result of chemical energy released due to the dilution process with a continuous phase. The whole process is done at a constant temperature without any phase transitions during emulsification. Kaur et al. developed a calcipotriol and clobetasol propionate nanoemulsion using a mix of Cremophor RH40, Capmul MCM C8 EP, and Labrafil 1944 CS as surfactant. The droplet size achieved was approximately 35.45 nm [164]. Sahu et al. studied the synergetic effect of tacrolimus and kalonji oil as a treatment of psoriasis. The resultant particle size from this study was 93.37 nm [166]. Ali et al. developed a turmeric oil with a mix of surfactants to produce a nanoemulsion with 20–200 nm particle size [167]. Alam et al. developed a nanoemulsion with betamethasone dipropionate paired with a mix of surfactants to produce a nanoemulsion with a particle size of 155.08 nm [168].

### 5.3. Self-Nanoemulsifying Drug Delivery Systems

Self-nanoemulsifying drug delivery systems (SNEDDS) use a technique of making an anhydrous complex containing drug, oil, and surfactant and introducing it into the aqueous phase of nanoemulsion. Mild agitation is generally used to incorporate the anhydrous complex into the aqueous phase to form the nanoemulsion. The time taken for the migration of hydrophilic components from the complex to the aqueous phase is dependent on the level of turbulence at the oil and aqueous interphase. As the migration occurs, the oil phase component gradually moves into the aqueous phase. As the oil phase moves into the aqueous phase, a continuous array of oil droplets form. The order of mixing the components does not significantly affect the formation of the nanoemulsion. By using this method, the resultant particles are predicted to have a globular shape with a size of less than 200 nm [191]. In the absence of surfactant, this process is also known as the Ouzo effect [192]. Ali et al. developed a clobetasol propionate nanoemulsion using the SNEDDS method with a mixture of Tween 20 as the surfactant. The mean droplet size for this nanoemulsion was approximately 85 nm [179]. Mohd et al. presented a glimepiride nanoemulsion using a mixture of Tween 80 as a surfactant. They managed to produce a nanoemulsion with a droplet size less than 200 nm [180]. Zhao et al. also used a similar method to produce an ibuprofen nanoemulsion with lemon essential oil as oil phase and Cremophor RH40 as surfactant. The range of droplet size for their nanoemulsion was 28 to 145 nm [181]. Bandyopadhyay et al. produced a valsartan nanoemulsion using long-chain triglycerides (LCTs) and medium-chain triglycerides (MCTs) as oil phase and Tween 40 and Tween 80 as emulgents [182]. Beg et al. also used the same lipids for paclitaxel and yielded a droplet size of 63.6–145 nm for MCT formulations and 84–133.5 nm for LCT formulations [193].

### 5.4. D-Phase Emulsification

**Figure 5:**
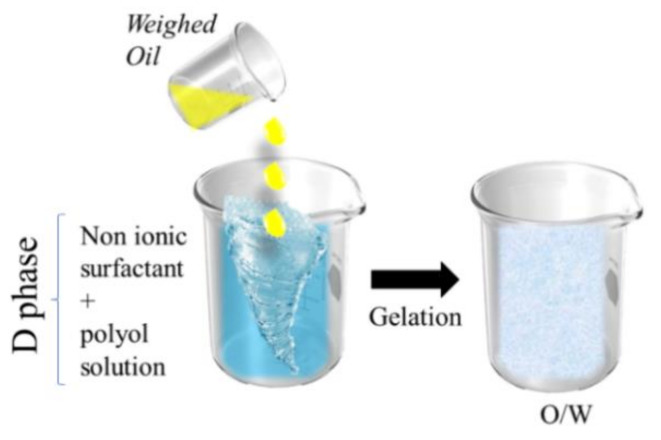
Illustration of D-phase emulsification. This is a self-drawn figure.

D-phase emulsification (DPE) is a method that utilizes nonionic surfactants. Generally, the procedure begins with mixing nonionic surfactant and polyol solution until it is in one phase, which is known as the D-phase (Figure 5). Into this D-phase, a certain amount of the oil phase is added drop by drop with stirring. By doing this, a clear gel comprising of both oil and surfactant is formed. The gel is then diluted with water to produce a fine O/W nanoemulsion [194]. Compared to the other low-energy methods, DPE requires less surfactant, does not use solvent, and conserves more energy than PIC [195]. In a study by Yukuyama et al., an olive oil nanoemulsion was developed using a hydrophilic surfactant polyethylene glycol monooleyl ether. The resultant particle size achieved was 20–30 nm [184]. Zhang et al. developed a mineral oil nanoemulsion using Tween 80-Span 80 mix as a surfactant. The particles formed from this study were approximately 52.35 nm [196]. In comparison to other low-energy methods, the DPE is a newly developed method that yields nanoparticles in the smallest range with regards to the minimal processing involved. A compilation of recent studies that successfully produced a nanoemulsion with low-energy methods can be found in Table 5.

## 6. The Recent Development of Nanoemulsions as a Treatment for Psoriasis

As mentioned in the previous section, nanoemulsions, is one of the ideal treatment options for psoriasis, can be formed through high-energy and low-energy emulsification methods. Low-energy methods are beneficial as they do not need expensive machinery for processing. However, the complexity of the low-energy methods lies in the choice of ingredients that make up the nanoemulsion. Usage of multipurpose ingredients could suggest a lower cost in upscale production. In that sense, oils used as a component in the nanoemulsion mix should be suited to their intended purpose (i.e., neem oil with skin healing properties can be used for the anti-psoriatic nanoemulsion). Another key component in the nanoemulsion is the surfactant of choice. With the growing trend of going ‘green’, biosurfactants are preferred as they are sourced from biological matter and can be mass produced easily and sustainably. 

To date, several anti-psoriatic nanoemulsions have been formulated as mentioned in the previous section. In the formulation done by Kaur et al., clobetasol propionate and calcipotriol was used as the drug of choice. They used an imiquimod-induced psoriatic BALB/c mice model, to study the anti-psoriatic activity. The results showed that the HaCaT cell layer had higher uptake of drug from the developed nanoemulsion and high penetration through the stratum corneum of the skin compared to the marketed drugs [164]. Besides, Musa et al. formulated their nanoemulsion using cyclosporin in a matrix consisted of virgin coconut oil and nutmeg oil. Their formulation was stable for up to 3 months and in the in-vivo skin analysis done on healthy volunteers, significant hydration was seen in the stratum corneum [165]. Sahu et al. used tacrolimus paired with Kalonji oil (black seed oil) for their nanoemulsion. Kalonji oil was chosen as it has anti-psoriatic properties. The results show an enhanced dermal bioavailability (4.33-fold) with significant in-vitro results. There was a reduction in serum cytokines and improvement in psoriatic condition in the in-vivo test, indicating the efficacy of the nanoemulsion [166]. In another study by Rajitha et al., Methotrexate incorporated nanoemulsion was produced paired with Chaulmoogra oil. This oil has been known to treat skin diseases in traditional medicine and provides an antioxidant and anti-inflammatory property to the developed nanoemulsion. The nanoemulsion was stable in the refrigerator for 3 months. The ex-vivo skin permeation studies done using a Franz diffusion cell model with porcine skin showed improved skin permeation and retention of drug in deep skin layers. The in-vivo studies done on imiquimod psoriatic model showed high anti-psoriatic efficacy, high skin retention, low serum and tissue accumulation when compared to mice treated with orally administered methotrexate [127]. Furthermore, a study by Ali et al. used a more natural approach by omitting the use of drug and just used turmeric oil as the anti-psoriatic agent. In the test carrageenan-induced paw edema model, it was found that there is a 70.35% inhibition of psoriasis [167]. In another study by Alam et al., the use of betamethasone dipropionate paired with a mix of Eucalyptus oil and Babchi oil was used to develop their nanoemulsion. The resulting nanoemulsion was able to inhibit edema up to 77.83% within 24 h in a carrageenan-induced hind paw edema model [168]. The use of clobetasol propionate and Eucalyptus oil nanoemulsion were also reported. In this study, Wistar rats were induced with dermatitis using 5% nickel sulphate and upon application with developed nanoemulsion, 84.15% inhibition was observed, twice more efficient than marketed cream after 12 h of treatment [172]. On the other hand, Kaur et al. developed an Amphotericin B nanoemulsion with sesame oil and soya bean oil as the oil phase. Amphotericin B is an antifungal that disrupts the synthesis of fungal cell wall because of its ability to bind to sterols, the main building block in cell wall synthesis. The antifungal property of nanoemulsion was evaluated using an in-vitro drug test and the best formulation from the lot gave 98.12% efficacy [170]. In a study by Eskandarii et al., evening primrose oil was used as the anti-psoriatic agent. This is another study which uses a natural approach in skin treatment. A 90-day stability test was successfully performed but no in-vivo or in-vitro works were carried out [171]. Somagani et al. developed a nanoemulsion with Aceclofenac and capsaicin as the active agents. This study uses a combination of nanoemulsion and nanomicelles systems known as nanomiemgel. An ex-vivo psoriatic mice model; imiquimod-induced psoriatic like plaque model was used and the result showed that the combination of nanosystems enhanced drug permeability [173]. Ahmad et al. had their take on anti-psoriatic nanoemulsions, using betamethasone dipropionate (BD) paired with Salmon fish oil. Fish oil was used to exploit its anti-inflammatory properties. The in-vivo anti-inflammatory activity was tested using a carrageenan edema induced model and the results of post-application of developed nanoemulsion was 85.22% inhibition compared to a 33.31% inhibition in placebo [174]. However, no clinical studies have been performed.

Based on the above studies, most anti-psoriatic nanoemulsions used a low-energy method to develop their nanoemulsion. This may be due to the natural oils used in the mixtures for their skin reparative properties. High-energy methods would normally implore high shear force and drastic temperature shifts during processing, hence a low-energy method was favoured to maintain the integrity of the natural oils. Furthermore, the developed psoriatic nanoemulsion generally use synthetic conventional surfactants. To achieve sustainable development, biosurfactants were introduced in this review as a suitable alternative. The recent studies on low-energy emulsification for psoriatic treatment with the particle size ranging from 9.6 to 220 nm have been summarised in Table 5, setting a benchmark for future nanoemulsion development.

## 7. Conclusions

Psoriasis is a long-term illness that requires a treatment that is safe for extensive repetitive use. Modern medicine has developed conventional drugs for treating this disease through oral consumption or topical application. However, these drugs decrease in efficiency over time as a result of the autoimmune responses of the body. Furthermore, long-term use of these drugs may cause other infirmities to crop up, worsening the situation. Since then, nano-drug delivery systems have been introduced, such as nanoemulsions, biopolymeric nanoparticles, liposomes, polymeric micelles, and dendrimer to improve the therapeutic efficacy and safety, as well as to enhance bioavailability. Amongst these delivery systems, nanoemulsion is advantageous, as it has a lower production cost and lesser process complexity. To produce a ‘greener’ nanoemulsion, biosurfactants are introduced to take the place of chemical surfactants. Biosurfactants are more cost-effective and sustainable for mass production as they can be harvested through bacterial progenation. This review also discusses the mechanism of action of psoriasis to understand the mode of action of conventional and natural therapies. Suggestive lists of oils and biosurfactants compiled showed that many variations of formulations can be made suited to different applications. In an anti-psoriatic nanoemulsion, biosurfactants of the class of glycolipids are most suitable to be used due to their skin enhancing properties. A selection of a suitable oil phase in nanoemulsion systems with anti-psoriatic properties with preclinical studies would be beneficial as to act in synergy with the active ingredient. In addition, the approach towards low-energy emulsification was highlighted as it conserves energy, reduces shear force, and maintains the integrity of active ingredients during processing. This review summarizes all the necessary information required to develop nanoemulsions as a promising drug carrier system with a more environmentally conscious approach. However, the success of the translation of nanoemulsion formulations is more dependent on the success of clinical studies that can scientifically prove their therapeutic potential. Lipid-based nanoemulsions and biosurfactants as stabilizers are seen as one of the alternative approaches to treating psoriasis, but they require clinical data for commercialization purposes.

## Figures and Tables

**Table 1:** List of current treatments available for psoriasis and its respective mechanisms.

		Anti-Psoriatic Drug/Material	Trade Name	Application	Mechanism	Reference
Conventional	Topical	Betamethasone/calcipotriol	Dovobet^®^ gel	Gel	These drugs inhibit cell proliferation and initiate cell differentiation on the affected skin. They also serve as immunomarkers.	[48,49,50]
		Betamethasone/calcipotriol	Xamiol^®^	Gel		
		Calcipotriol	Calcipotriol^®^ ointment	Ointment		
		Calcipotriol	Dovonex^®^ ointment	Ointment		[51]
		Coal tar	Capasal^®^	Shampoo	These drugs inhibit cell proliferation and initiate cell differentiation on the affected skin. They also serve as immunomarkers.	[52,53]
		Coal tar, coconut oil, salicylic acid, sulfur	Cocois^®^	Ointment		[52]
		Coal tar	Carbo-Dome^®^	Cream		[54]
		Coal tar	Exorex^®^	Lotion		[55]
		Coal tar	Polytar^®^ plus liquid	Solution		[56]
		Coal tar	Psoriderm^®^ cream	Cream		[57]
		Coal tar, coconut oil, salicylic acid, sulfur	Sebco^®^	Ointment		[58]
		Concentrate cetrimide, phenylethyl alcohol, undecenoic acid (antifungal and antibacterial)	Ceanel^®^	Shampoo	This drug demonstrates antibacterial activity. As the drug is hydrated, small inactive anions and complex cations are dissociated, activating its bactericidal properties.	[59,60]
		Tacalcitol	Curatoderm^®^	Ointment	These drugs regulate cell apoptosis, differentiation, proliferation, and immunomodulation.	[59,61]
		Tacalcitol	Curatoderm^®^ lotion	Lotion		[62]
		Benzalkonium chloride	Dermax^®^ therapeutic shampoo	Shampoo	This drug exhibits antifungal and antibacterial activity.	[63,64]
		Dithranol	Dithrocream^®^	Cream	These drugs inhibit hyperproliferation of keratinocyte and granulocyte function. They may also help to suppress the immunoresponse triggered by psoriasis.	[63]
		2% Dithranol	Dithrocream^®^	Cream		[63]
		Dithranol	Micanol^®^	Cream		[65,66]
	Systemic	Methotrexate	Otrexup™, Rasuvo^®^, Rheumatrex^®^, and Trexall™	Subcutaneous injection or oral	This drug inhibits dihydrofolate reductase production, which offsets purine synthesis. This may also lead to lymphocyte apoptosis.	[66]
		Cyclosporine	Neoral, Sandimmune, and Gengraf	Oral	This drug inhibits production of calcineurin, leading to a reduction in IL-2 levels.	[67]
		Acitretin	Soriatane and Neotigason	Oral	This drug helps normalize the proliferation of keratinocyte and differentiation by binding to the retinoid receptor.	[68]
		Fumarate	Tecfidera	Oral	This drug regulates intracellular glutathione, Nrf2, HIF-1α, and NF-κB, and by doing so creates a shift from pro-inflammatory Th1/Th17 response to an anti-inflammatory/regulatory Th2 response.	[69]
		Apremilast	Otezla, Aplex	Oral	This drug inhibits PDE4, thereby increasing the level of intracellular cAMP in immune and non-immune cells, reducing the inflammation.	[70]
		Etanercept	Enbrel	Subcutaneous injection	This drug is a dimeric human fusion protein that is a TNF-α antagonist. TNF-α is the pro-inflammatory cytokine in pathogenesis.	[71]
		Infliximab	Remicade	Intravenous	This drug is a chimeric IgG1κ monoclonal antibody that binds to soluble and transmembrane forms of TNF-α, another TNF-α antagonist.	[72]
		Adalimumab	Humira, Mabura, Exemptia	Subcutaneous injection	This drug is a human monoclonal antibody that is yet another TNF-α antagonist.	[73]
		Certolizumab	Cimzia	Subcutaneous injection	This drug is a humanized antigen-binding fragment (Fab) of monoclonal antibody TNF-α conjugated to polyethylene glycol.	[74]
		Ustekinumab	Stelara	Subcutaneous injection	The drug is a human IgG1k monoclonal antibody that specifically binds to p40 protein subunit that is required for IL-12 and IL-23 cytokines IL-12/IL-23 p40 functionality.	[75]
		Tildrakizumab	Ilumya, Ilumetri	Subcutaneous injection	The drug is a humanized IgG1κ, which inhibits IL-23 selectively by binding to its p19 subunit.	[76]
		Guselkumab	Tremfya	Subcutaneous injection	The drug is a human immunoglobulin G1 lambda (IgG1λ) monoclonal antibody, inhibiting IL-23 selectively by binding to its p19 subunit.	[77]
		Risankizumab	Skyrizi	Subcutaneous injection	The drug is a humanized IgG1 monoclonal antibody that inhibits IL-23 by binding to its p19 subunit.	[78]
		Secukinumab	Cosentyx	Subcutaneous injection	This drug is a human IgG1κ monoclonal antibody that is a IL-17A antagonist.	[79]
		Ixekizumab	Taltz	Subcutaneous injection	This drug is a humanized, immunoglobulin G4κ monoclonal antibody that binds selectively to IL-17A and thereby neutralizes it functionality.	[80]
		Brodalumab	Siliq, Kyntheum	Subcutaneous injection	This drug is a human monoclonal IgG2 antibody that acts against IL-17RA.	[81]
Natural		Bee venom	-	Venom acupuncture	The venom has an analgesic and anti-inflammatory effect.	[82]
		Aloe vera (aloesin)	-	Gel	Aloesin initiates the upregulation of cytokines and growth factor (IL-1β, IL-6, TGF-β1, and TNF-α) release from macrophages. This enhances the angiogenesis in endothelial cells. It also accelerates wound healing in mice model by activating MAPK and Smad signalling proteins.	[83]
		Turmeric (curcumin)	-	Powder	Curcumin inhibits the proliferation of IMQ-induced differentiated HaCaT cells (psoriatic-like cells) by downregulation of pro-inflammatory cytokines, IL-17, TNF-α, IFN-γ, and IL-6.	[84]
		Oregon grape (berberine)	-	Ointment/cream	Berberine inhibits 5-lipoxygenase and lipid peroxidation in liposomes, as well as immune markers ICAM-1, CD-3, and HLA-DR, and keratins 6 and 16, resulting in an antiproliferative effect on human keratinocytes.	[85]
		Propolis	-	Ethanolic extract	Propolis prevents the release of prostaglandins and leukotrienes, and it decreases neutrophil infiltration into the skin.	[86]
		Neem (azadirachtin)	-	Soap	Azadirachtin modulates the activity of transcription factors NF-κB and cell apoptosis.	[87]

**Table 2:** Suggestive list of oil phases to be used in nanoemulsion.

	Oil Phase	Source	Matrix	Particle Size (nm)	Ref.
Oils	Fish oil	Fishmeal and ensilage	Carp viscera fish oil, chitosan, gelatine	96.5–166.5	[115]
	Castor oil	Seed of ricinus communis	Castor oil, mineral oil, sorbitan monostearate, polysorbate 80	234	[116]
	Corn oil	Germ of corn	Corn oil, glycerine, Tween 80^®^, Span	250	[117]
	Coconut oil	Kernel of coconut	Tween 80^®^	18–62	[118]
	Evening primrose oil	Seeds of oenothera biennis	Virgin coconut oil, mangosteen peels, propylene glycol, Tween 20/Span 20	202- 215	[119]
	Linseed oil	Seeds of linum usitatissimum	Evening primrose oil, sorbitan oleate, polysorbate 80	20.34–314.77	[120]
	Olive oil	Olive fruit	Linseed oil, perilla oil, cremophor rh40, Span 80	39.22	[121]
	Palm oil	Mesocarp of palm fruit	Olive oil, Tween 80	275.5	[122]
	Sunflower oil	Seeds of sunflower	Palm oil, curcumin, Tween 80	100–500	[123]
	Neem oil	Kernel of neem seed	Sunflower oil, Tween 80, sorbitol	10 to 100	[124]
	Babchi oil	Seed of Psoralea corylifolia	Neem oil, Tween 20	169–228	[125]
	Grapeseed oil	Seeds of grapes	Babchi oil, cat, Tween 80, transcutol-p, distilled water	137.49-163.82	[126]
	Chaumogra oil	Seeds of hydnocarpus flacourtiaceae	Grapeseed oil, Tween 80, PEG 400	36.33-49.11	[127]
	Tea tree oil	Leaves of Melaleuca alternifolia	Chaulmoogra oil, methotrexate, Tween 80, ethanol	166.7–188.2	[128]
	Argan oil	Kernel of argan fruit	Tea tree oil, Tween 80, propylene glycol	129–231	[129]
	Sesame seed oil	Sesame seeds	Argan oil, sorbitan monolaurate, water	62.2	[130]
	Jojoba oil	Seeds of simmondsia chinensis	Sesame seed oil, Tween 80, sorbitan monooleate	30–300	[131]
Waxes	Carnauba wax	Leaves of Copernicia prunifera palm	Jojoba oil, sorbitan oleate, polysorbate 80	69 ± 0.63	[132]
	Paraffin wax	Petroleum	Carnauba wax, soybean lecithin, Tween 80	160.9	[133]
	Bees wax	Bees	Paraffin wax, sodium	95.72 ± 9.63	[134]
	Candelilla	Leave of candelilla shrub	Lauryl sulphate, distilled water	50	[135]
	Rice bran wax	Bran oil of rice (Oryza sativa)	Beeswax, carnauba wax, Polaxamer 407, Tween 80	92.56–94.52	[136]

**Table 3:** Suggestive list of biosurfactants to be used in nanoemulsion.

Biosurfactant	Class	Source	Organism	Cosmetic Properties	Chemical Structure	CMC	Reference
Rhamnolipid	Glycolipid	Bacteria	Pseudomonas aeruginosa	This biosurfactant is a good emulsifying agent. It also has antimicrobial activity against Staphylococcus aureus, Bacillus cereus, Micrococcus luteus, and monocytogenes. It also is known for its skin-repairing abilities by reduced fibrosis.	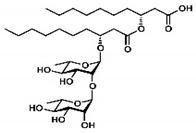	5–50 mg/L	[142]
Mannosylerthriol lipid	Glycolipid	Fungi	Pseudozyma and Ustilago	This biosurfactant has antiaging properties, particularly in the recovery of skin cells that have been damaged by UV radiation. It also exhibits antimicrobial activities.	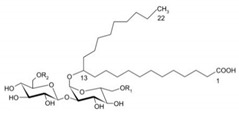	1.50–100 mg/L	[143]
Trehalose lipid	Glycolipid	Bacteria	Mycobacterium, Rhodococcus, Arthrobacter, Nocardia, and Gordonia	The biosurfactant can reduce the interfacial tension and increase pseudosolubility of hydrophobic compounds. It also exhibits antibacterial activity against Escherichia coli, Vibrio harveyi, Proteus vulgaris, and Candida albicans. The mode of biocidal action is by antiadhesion. It also has hydration and restructuring properties.	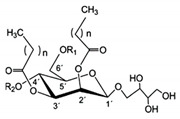	37 mg/L	[144]
Sophorolipid	Glycolipid	Fungi	Rhodotorula bogoriensis	This biosurfactant has antiaging properties, specifically antiwrinkling.	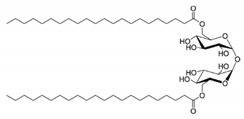	9.5 mg/L	[145]
Cellobioselipid	Glycolipid	Fungi	Pseudozymafusiformata, Cryptococcus humicola, Sclerotinia sclerotiorum, Phomopsis helianthi, Ustilago maydis	This biosurfactant can facilitate the dissolution and consumption of organic hydrophobic compounds. It is also known for its antifungal properties.	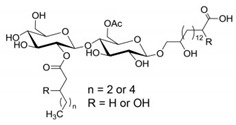	2 × 10^−5^ M (pH 4.0), 3.3 × 10^−5^ M (pH 4.0)	[146]
Emulsan	Polymeric biosurfactant	Bacteria	Acinetobacter calcoaceticus	This biosurfactant is used in the removal of residual oil in oil tanks.	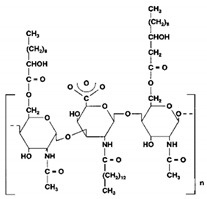	no information available	[147]
Surfactin	Lipopeptides and oligopeptides	Bacteria	Bacillus subtilis	This biosurfactant inhibits fibrin clot formation and has antibacterial properties.	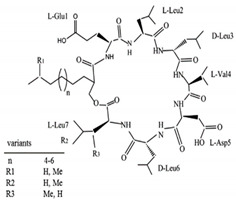	9.4 µM (pH 8.7)	[148,149]
Viscosin	Lipopeptides and oligopeptides	Bacteria	Pseudomonas sp.	This biosurfactant has antifungal activity against Aspergillus fumigatus and Batrachochytrium dendrobatidis.	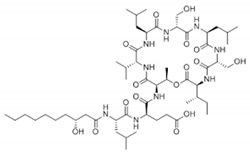	54 mg/L	[150,151]
Amphisin	Lipopeptides and oligopeptides	Bacteria	Pseudomonas sp.	This biosurfactant has antifungal activity.	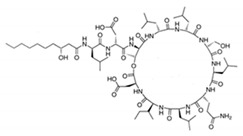	54 mg/L	[152]
Tolaasin	Lipopeptides and oligopeptides	Bacteria	Pseudomonas	This biosurfactant has antibacterial activity against Bacillus megaterium.	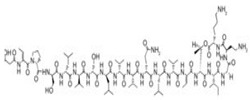	no information available	[153]
Syringomycin	Lipopeptides and oligopeptides	Bacteria	Pseudomonas sp.	This biosurfactant has fungicidal activity against various yeast, Aspergillus species, Candida, Cryptococcus, and Geotrichum.	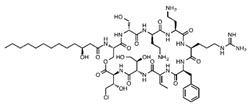	1.25 mg/mL	[154]
Serrawettin	Lipopeptides and oligopeptides	Bacteria	Serratia marcescens	This biosurfactant has slight or almost insignificant antibacterial activity.	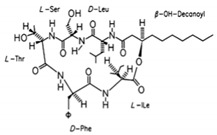	no information available	[155]

**Table 4:** Colloidal properties of common synthetic surfactants and biosurfactants.

Surfactant	Type	Chemical Grouping	CMC	Surface Tension (mN/m)	Reference
Biosurfactant					
Rhamnolipid	Anionic/nonionic	Glycolipid	5 mg/L	25–40	[156]
Mannosylerthriol lipid	Anionic	Glycolipid	1.50–100 mg/L	30–33	[157]
Trehalose lipid	N/A	Glycolipid	1.04–37 mg/L	34	[158]
Sophorolipid	Anionic/nonionic	Glycolipid	9.5 mg/L	30–43	[159]
Cellobioselipid	N/A	Glycolipid	20 µM (pH 4.0), 33 µM (pH 4.0)	37 in 0.1 M NaHCO_3_ 23 °C	[159]
Surfactin	Zwitterionic	Lipopeptides and oligopeptides	20–130 mg/L	27.9–36	[160]
Viscosin	N/A	Lipopeptides and oligopeptides	54 mg/L	28	[151]
Amphisin	N/A	Lipopeptides and oligopeptides	54 mg/L	32	[161]
Syringomycin	N/A	Lipopeptides and oligopeptides	1.25 mg/mL	33	[162]
Synthetic					
Alkyl polyglucosides	Nonionic	Glycolipid	67–87 mg/L	28–29	[163] ^p^
Dodecyl maltoside	Nonionic	Glycolipid	0.2 mM (102 mg/L)	35.5	
SDS	Anionic	Organic sodium salt	7–8 mM (2–3 g/L)	38	
Hexa-ethyleneglycol mono n-dodecyl ether (C12E6)	Nonionic	Alcohol ethoxylate	0.087 mM (39 mg/L)	33	
Tetraethylene glycol monodecyl ether (C10E4)	Nonionic	Glycol ether	0.68 mM (227 mg/L)	30	
Triton X-100	Nonionic	Poly (ethylene glycol) derivative	140 mg/L	30–31	
Tween/polysorbate 80	Nonionic	Polysorbates	17 mg/L	42	
Tween/polysorbate 20	Nonionic	Polysorbates	70 mg/L	30	

^p^ Note. Partially adapted from “Biosurfactants and surfactants interacting with membranes and proteins: Same but different?” by Daniel E. Otzen, 2017, *Biochim Biophys Acta Biomembr*, 1859(4):639–649.

**Table 5:** List of recent studies on low-energy nanoemulsification for psoriatic treatment and other applications.

	Active Ingredient	Oil	Surfactant	Matrix	Particle Size (nm)	NE Method	Ref
**Psoriasis Treatment**	Clobitasol propionate and calcipotriol	Capmul MCM C8 EP	Cremophor RH 40	Capmul MCM C8 EP, Cremophor RH 40, Labrafil 1944 CS	35.45 ± 2.68	Spontaneous emulsification	[164]
	Cyclosporin	Virgin coconut oil, nutmeg oil	Polysorbate 80 (Tween 80)	Virgin coconut oil, nutmeg oil, Tween 80, Xanthan gum, Phenonip	100–200	Standard sample-dilution	[165]
	Tacrolimus and Kalonji oil	Tacrolimus and Kalonji oil	Cremophor RH 40	Tacrolimus and Kalonji oil, Cremophor RH 40, polyethylene glycol 400	93.37 ± 2.58	Spontaneous emulsification	[166]
	Methotrexate	Chaulmoogra oil	Tween 80	Chaulmoogra oil, Tween 80, ethanol	34 (with negative surface charge)	Low-energy self-emulsification	[127]
	Turmeric oil	Turmeric oil	Tween 20/ Tween 80/ Labrasol/ Lecithin	Turmeric oil, Tween 20/ Tween 80/ Labrasol/ Lecithin, isopropyl alcohol	20–200	Spontaneous emulsification	[167]
	Betamethasone dipropionate (BD)	Eucalyptus oil and babchi oil	Labrasol, Tween 80, Tween 20, Brij35	Eucalyptus oil and babchi oil, labrasol/ Tween 80/ Tween 20/ Brij35, Ethanol/ propanol/ PEG 200/ Glycol/ Capryol and Pleurol oleic	155.08	Spontaneous emulsification	[168]
	Amphotericin B (amb)	Sefsol-218 oil	Tween 80	Sefsol-218 oil, Tween 80, Transcutol-P	97.04 ± 7.4	Slow and spontaneous emulsification titration	[169]
	Amphotericin B	Sesame oil or soya bean oil	Tween 80	Sesame oil or soya bean oil, Tween 80, glycerol, α-tocopherol (antioxidant)	-	Hot homogenization	[170]
	Evening primrose oil	Evening primrose oil	Tween 80	Evening primrose oil, Tween 80	164	-	[171]
	Clobetasol propionate	Eucalyptus oil	Tween 20	Eucalyptus oil, Tween 20, ethanol	10–200	Aqueous phase titration	[172]
	Aceclofenac and capsaicin	Olive oil and miglyol	Polysorbate 80	Olive oil and miglyol, Tween 80, Transcutol	200–220	High-pressure homogenization	[173]
	Betamethasone Dipropionate (BD)	Salmon fish oil	Tween 80	Salmon fish oil, Tween 80, Transcutol P, carbopol 971 (gelling agent)	129.89	Aqueous phase titration	[174]
**Other applications**	Hexadecane and oleic acid mixture	Hexadecane	Oleyl ammonium (cationic surfactant)	Hexadecane, Oleylammonium (cationic surfactant)	Micelle size (µm range)	Phase inversion composition (PIC)	[175]
	Fisetin	Miglyol 812N	Tween 80	Miglyol 812N, Tween 80, Lipoid E80	153 ± 2	Phase inversion temperature (PIT)	[176]
	Lemon oil	Lemon oil	Tween 40	Lemon oil, Tween 40	9.6 and 11.1	Phase inversion temperature (PIT)	[177]
	Cinnamon oil	Cinnamon oil and medium-chain triglyceride (MCT)	Tween 80	Cinnamon oil, medium-chain triglyceride (MCT), Tween 80	101	Phase inversion temperature (PIT)	[178]
	Celecoxib	Eucalyptus oil	Tween 20	Eucalyptus oil, Tween 20, ethanol	85.31–86.01	Self-nanoemulsifying drug delivery systems (SNEDDS)	[179]
	Glimepiride	Miglyol 812	Tween 80	Miglyol 812, Tween 80, PEG 400	<200	SNEDDS	[180]
	Ibuprofen	Lemon essential oil	Cremophor RH40	Lemon essential oil, Cremophor RH40, Transcutol HP	40–99	SNEDDS	[181]
	Valsartan	Lauroglycol FCC and Capmul MCM L8	Tween 40	Lauroglycol FCC and Capmul MCM L8, Tween 40, Tween 80	-	SNEDDS	[182]
	Glibenclamide	Labrasol, HCO-60	Tween-80	Labrasol, HCO-60, Tween-80, Cremophor-EL	39.1	SNEDDS	[183]
	Olive oil	Olive oil	Polyethylene glycol monooleyl ether (Oleth-20)	Olive oil, polyethylene glycol monooleyl ether (Oleth-20)	20–30	D-phase emulsification	[184]

## Data Availability

Not applicable.
